# SOX2 Promotes the Epithelial to Mesenchymal Transition of Esophageal Squamous Cells by Modulating Slug Expression through the Activation of STAT3/HIF-α Signaling

**DOI:** 10.3390/ijms160921643

**Published:** 2015-09-08

**Authors:** Hui Gao, Chunyuan Teng, Wenjing Huang, Jianjun Peng, Chunbo Wang

**Affiliations:** 1 Department of Pharmacology, Medical College Qingdao University, Qingdao 266071, China; E-Mail: amethystgh@126.com; 2 Department of Gastroenterology, Qingdao Hiser Medical Center, Qingdao 266033, China; E-Mail: Chunyuanteng@126.com; 3 Department of Paediatrics, the Affiliated Hospital of Medical College Qingdao University, Qingdao 266003, China; E-Mail: wenjinghuang15@163.com; 4 College of Life Sciences, Chongqing Normal University, Chongqing 401331, China

**Keywords:** SOX2, esophageal squamous cell carcinoma, Slug, metastasis, EMT (epithelial to mesenchymal transition)

## Abstract

The transcription factor sex determining region (Y SRY)-box 2 (SOX2) is known to play a crucial role in the maintenance of self renewal or pluripotency of undifferentiated embryonic and neuronal stem cells. An elevated expression of SOX2 has been correlated with poor prognosis of esophageal squamous cell carcinoma (ESCC). We sought to investigate the mechanism(s) by which SOX2 modulates the ESCC metastasis. The SOX2 coding DNA sequence was inserted into pCMV vector and stably transfected in ESCC cells (Eca-109). The effect of SOX2 over expression was evaluated on cell migration, invasion and epithelial to mesenchymal transition (EMT). We also measured the expression of Slug to explore if this transcription factor is involved in SOX2-mediated regulation of cell migration/invasion and EMT. In addition, we determined the role of STAT3/HIF-1α to further probe the mechanism of SOX2-mediated metastasis via Slug. Our results demonstrated that SOX2 over expressing Eca-109 cells showed an enhanced cell migration/invasion. Moreover, these cells exhibited the EMT characteristics, that is, a significantly suppressed expression of the epithelial cells marker with a concomitant enhancement of those of the mesenchymal markers. An increased expression of Slug in SOX2 over expressing cells suggested the involvement of this transcription factor in SOX2-regulated metastasis. Whereas the expressions of STAT3/HIF-1α were found to be up-regulated in SOX2 expressing cells, blockade of these transcription factors resulted in the inhibition of Slug expression at both protein and mRNA levels. Conclusion: These results suggest that SOX2 promoted the metastasis of ESCC, at least in part, by modulating Slug expression through the activation of STAT3/HIF-1α signaling.

## 1. Introduction

Esophageal cancer is one of the major health problems and the sixth leading cause of cancer-related deaths worldwide [1]. Whereas, in the Western countries, adenocarcinoma is the most prevalent type of ESCC (esophageal squamous cell carcinoma), 90% of the esophageal cancers in the region stretching from northern Iran to North-Central China are known to arise from the squamous cells [2]. ESCC has been associated with grim prognosis because, by the time it is diagnosed, most the patients are presented with locally progressed disease such that less than 40% of them have a resectable tumor [3]. The late diagnosis of ESCC is associated with an advanced stage, and high rate of metastasis. As a consequence, despite the use of multiple therapeutic strategies, the five-year survival rate is low (15%–20%) [4]. Therefore, It is required that biological mechanisms underlying the ESCC metastasis be unraveled so as to improve the disease prognosis.

SOX2 is a 317 amino acids protein that belongs to the family of SOX (SRY-related high mobility group box) transcription factors is encoded by a gene located on chromosome 3q26.3-q27 [5,6]. A number of studies have been conducted to determine the functional role of SOX2 in the induction and maintenance of pluripotency or self-renewal of embryonic stem cells [7,8,9]. Clinical findings suggesting an association between alterations in *SOX2* gene and developmental maladies have been evidenced. For instance, anophthalmia-esophageal-genital (AEG) syndrome characterized by an abnormal development of ectodermal and endodermal tissues, has been found to occur as a result of a heterozygous mutation in *SOX2* gene [10]. In a variety of human malignancies, SOX2 has been associated with increased cell proliferation, apoptosis, invasion and metastasis [11]. In the case of ESCC, an elevated expression of SOX2 has also been reported to result in the promotion of proliferation and growth of tumor cells [12,13]. However, the role of SOX2 in ESCC metastasis has not been completely elucidated.

Metastasis is commonly associated with the progression of malignancy and it involves multiple sequential steps, including invasion of cancer cells into the surrounding tissues, penetration of the lymphatic/blood vessel walls, survival in circulation, re-penetration through the vessels, and growth into a macroscopic secondary tumor in the distant organs [14]. The epithelial to mesenchymal transition (EMT) that normally occurs during embryonic development and cancer metastasis is known to be regulated by well coordinated actions of a number of proteins that are strategically localized in nuclear and cytosolic compartments [15]. As a result of EMT, the epithelial cells lose their inter cellular junctions and polarity, which renders them mobile and facilitates their invasion into the surrounding tissues or distant organs [16,17]. In this study, we are able to show that SOX2 over expressing (Eca-1) cells exhibited an enhanced migration/invasion and EMT. These processes induced by SOX2 were mediated through the modulation of Slug via STAT3/HIF-α activation.

## 2. Results

### 2.1. SOX2 over Expression Promotes the Migration and Invasion of Eca-109 Cells

**Figure 1:**
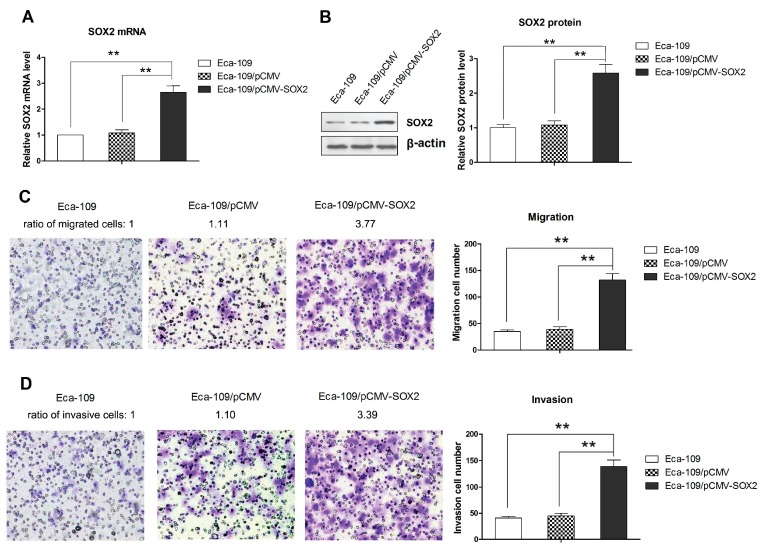
Over expression of SOX2 in Eca-109 cells and effect of SOX2 over expression on migration and invasion. (**A**) The SOX2 mRNA expression measured by qRT-PCR; (**B**) Western blot showing the SOX2 protein expression; (**C**) Cell migration assessed by Transwell chamber assay without Matrigel; (**D**) Cell invasion assessed by Transwell with Matrigel (200× magnification). Data are represented as mean ± SD of three separately conducted experiments. ** indicates *p* < 0.01. The Western blot and the images of Transwell chamber assay represent three independently conducted experiments.

The levels of SOX2 mRNA and protein expressions in stably transfected Eca-109 cells were confirmed by qRT-PCR and Western blot analysis, respectively. Our results showed that Eca-109/pCMV-SOX2 cells expressed significantly higher levels of SOX2 than the Eca-109/pCMV or parental Eca-109 cells (Figure 1A,B). The *in vitro* cell migration and invasion were assessed by determining the number of cells that migrated across the membrane without or with Matrigel, respectively, after a 24-h incubation. As shown in Figure 1C, SOX2 over expressing cells (Eca-109/pCMV-SOX2) exhibited a significantly increased migratory capability compared to those with control vector or the parental Eca-109 cells. The Eca-109 cells over expressing SOX2 showed a significant increase in cell invasion compared to the controls (Figure 1D). Collectively, these results suggest that SOX2 positively regulates the migration and invasion of Eca-109 cells.

### 2.2. SOX2 Promotes the Process of EMT in Eca-109 Cells

**Figure 2:**
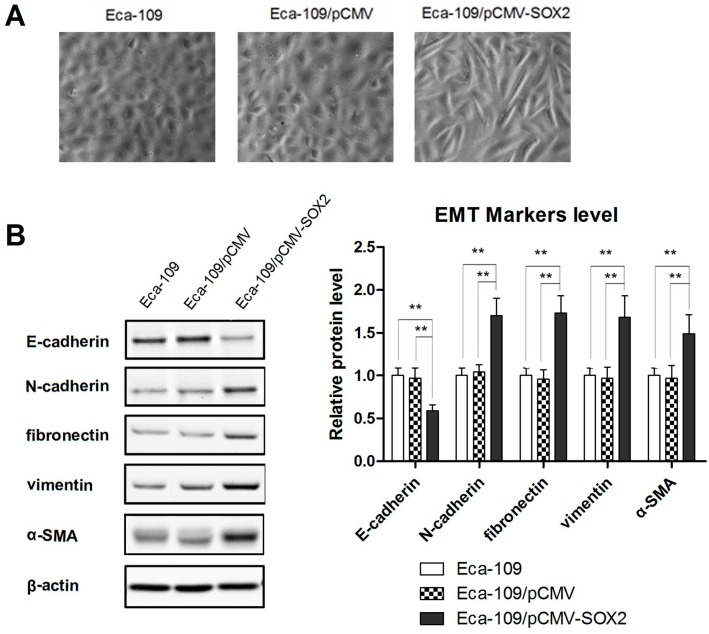
SOX2 over expression promotes the EMT process. (**A**) Phase-contrast microscopy images showing the effect of SOX2 over expression on cell morphology; (**B**) Western blot showing the effect of SOX2 on the expressions of epithelial and mesenchymal cells markers (E-cadherin, N-cadherin, vimentin, fibronectin and α-SMA). Data are represented as mean ± SD of three independently conducted experiments. ** indicates *p* < 0.01. The microscopic images (20× magnification) and Western blots are representatives of three independent experiments (The other two western blot image were provided as Figure S1).

In the process of EMT, specific morphological changes leading to loss of cell to cell tight contact occur in the epithelial cells. The transformed cells acquire characteristics of mesenchymal cells, which facilitates their mobility and hence promotes invasiveness into the surrounding tissue and/or distant organs. The epithelial protein E-cadherin is down regulated and mesenchymal proteins, such as vimentin and N-cadherin are up-regulated. Therefore, the morphological changes were first examined. As shown in Figure 2A, instead of a uniformly scattered pattern of the normal cells, the SOX2 over expressing cells exhibited a spindle- or star-like morphology characteristic of squamous cells. In order to further understand the role of SOX2 in metastasis of ESCC, we examined the expression of EMT markers by Western blotting. Whereas the expression of epithelial marker E-cadherin was significantly decreased in SOX2 over expressing cells, those of mesenchymal markers, N-cadherin, vimentin, fibronectin and α-SMA, were found to be increased markedly (Figure 2B), clearly indicating the occurrence of EMT.

### 2.3. SOX2-Promoted EMT Is Dependent on the Activity of Slug

**Figure 3:**
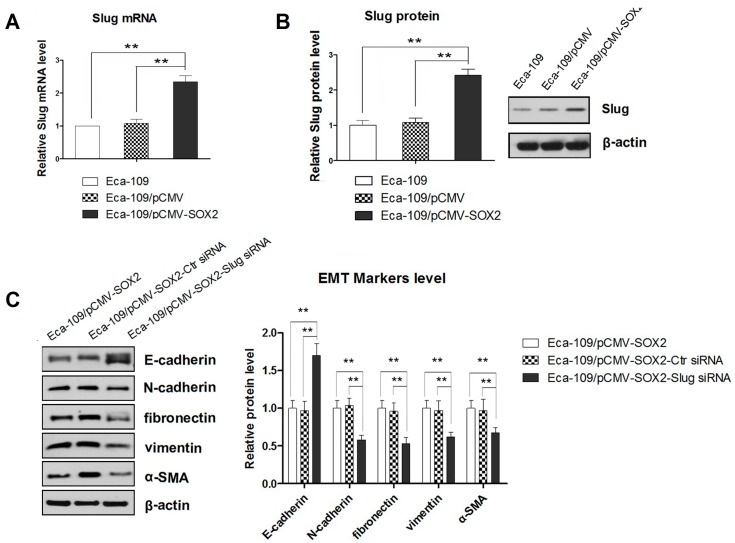

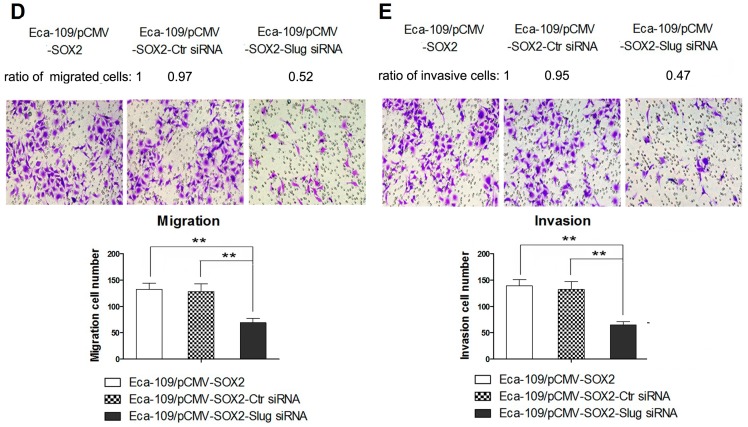
(**A**) The Slug mRNA expression measured by qRT-PCR; (**B**) Western blot showing the expression of Slug protein; (**C**) Western blot showing the effect of Slug knockdown on the expression of epithelial and mesenchymal markers in SOX2 overexpressing cells; (**D**,**E**) Effect of Slug knockdown on cell migration and invasion assessed by Transwell chamber assay without or with Matrigel, respectively (200× magnification). Data are represented as mean ± SD of three independently conducted experiments. ** indicates *p* < 0.01. Transwell chamber assay images and Western blots represent three independent experiments (The other two Western blot image were provided as Figure S2).

Slug has previously been shown to play a vital role in metastasis of ESCC and therefore it has been indicated to be a predictor of poor prognosis [18]. Therefore, we examined the mRNA and protein expressions of Slug in SOX2 over expressing cells by qRT-PCR and Western blot analysis, respectively. Our results showed that SOX2 over expression in Eca-109 cells was correlated with increased expression of Slug (Figure 3A,B). In order to further confirm the involvement of Slug in SOX2-mediated EMT, we incubated the SOX2-overexpressing Eca-109 cells with siRNA to knock down the Slug. As a result, the Slug expression was found to be substantially decreased (*p* < 0.01). Next, we examined the changes in the expressions of epithelial and mesenchymal markers in the cells treated with Slug-targeting siRNA. The Western blots showed a significant increase in the expression of epithelial marker E-cadherin and an equally marked decrease in the levels of the mesenchymal markers N-cadherin, fibronectin, vimentin and α-SMA (*p* < 0.01) (Figure 3C). These results demonstrating the blockade of SOX2-promoted EMT with down regulation of Slug, confirmed the involvement of Slug in this process of transition. Furthermore, suppressing the Slug expression resulted in compromised migration and invasion of the SOX2 expressing cells (Figure 3D,E), clearly indicating that Slug acted as an essential mediator in SOX2-induced cell migration and invasion.

### 2.4. STAT3/HIF-1α Signaling Is Involved in SOX2-Induced Up-Regulation of Slug Expression in Eca109 Cells

**Figure 4:**
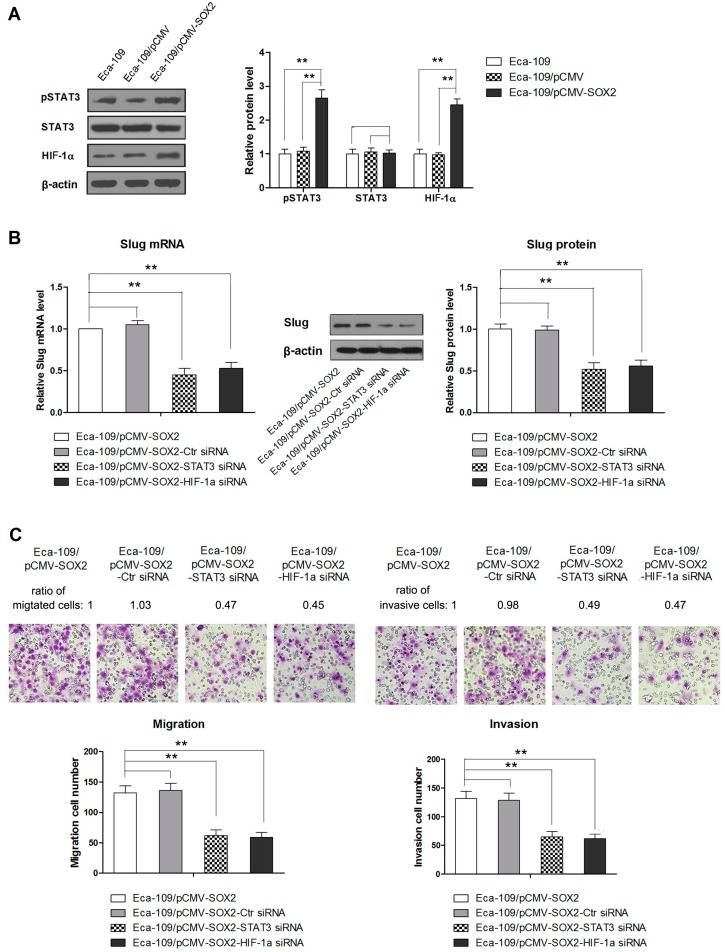

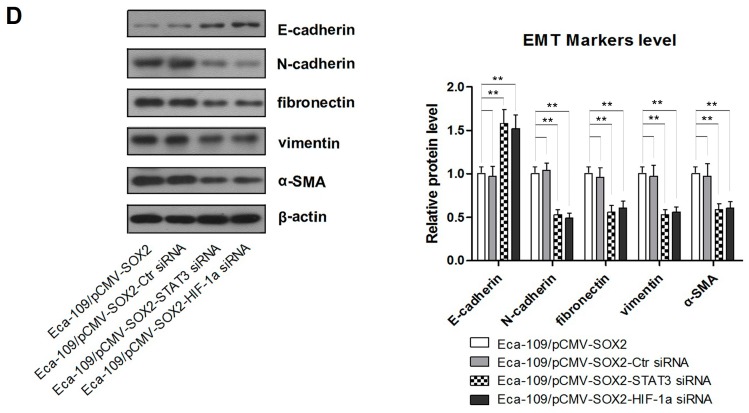
(**A**) Western blot showing the expression of STAT3/HIF-1α in Eca109/pCMV-SOX2 cells; (**B**) Western blot showing the effect of STAT3 or HIF-1α knockdown with specific siRNA on Slug expression; (**C**) The effect of STAT3 or HIF-1α knockdown with specific siRNA on migration and invasion of Eca109/pCMV-SOX2 cells evaluated by Transwell assay; (**D**) Western blots showing the effect of STAT3 or HIF-1α knockdown with specific siRNA on EMT (200× magnification). Data are represented as mean ± SD of three independently conducted experiments, ** indicates *p* < 0.01. The images of Transwell chamber assay and Western blots are representatives of three independent experiments.

STAT3/HIF-1α axis has been implicated in a number of cellular functions including tumor growth and metastasis [19,20]. Meanwhile, HIF-1α has been known to regulate expression of Slug in a variety of cell models [21,22]. Therefore, we asked the question whether STAT3/HIF-1α pathway is involved in SOX2-mediated up-regulation of Slug in Eca-109 cells. Compared to the controls, the SOX2 over expressing cells, Eca-109/pCMV-SOX2, showed a dramatic increase in the levels of pSTAT3 and HIF-1α, indicating the implication of these transcription factors in the regulation of metastasis (Figure 4A). Next, we investigated the role of STAT3/HIF-1α in SOX-2-induced EMT by using STAT3 and HIF-1α targeting siRNAs. Our results showed that blockade of both STAT3 and HIF-1α caused a significant suppression of Slug expression (Figure 4B). In addition, the migration and invasion of these cells were significantly reduced after targeted inhibition of STAT3 or HIF-1α with their respective siRNAs (Figure 4C). Moreover, in the presence of these siRNAs, the expression of epithelial marker was significantly enhanced, whereas those of mesenchymal markers were inhibited suggesting a blockade of SOX2-mediated EMT (Figure 4D). Taken together, these results have demonstrated that activation of STAT3/HIF-1α signaling is involved in the SOX2-induced Slug-mediated EMT.

## 3. Discussions

The human transcription factor SOX2, discovered in 1994, plays a crucial role in the maintenance of self-renewal or pluripotency of the embryonic stem cells (ESCs) [6]. Aberrant expression of SOX2 has been identified in several human tumors including lung, pancreatic, breast, ovarian, hepatocellular and head/neck [28,29,30,31]. However, the pattern of SOX2 expression and its correlation with clinical/pathological outcomes of various cancers is highly controversial. In renal cell carcinoma and gastric cancer, the expression of SOX2 has been found to be reduced [32,33]. A few studies have revealed an association of SOX2 expression with a favorable prognosis in non-small cell lung cancer, gastric cancer and renal cell carcinoma [32,33,34]. In contrast, others have related it with worse clinical outcomes, including recurrence, lymph node and distant metastasis in a variety of human malignancies [35,36,37]. In the case of esophageal cancer, an elevated expression of SOX2 has been associated with enhanced proliferation and growth of ESCC tumor cells [12,13]. On the contrary, loss of SOX2 expression has been correlated with a poor prognosis in patients of esophageal adenoma [38]. It is most likely that the role of SOX2 in the tumorigenesis depends on histological and organizational origin of the cells.

Previous studies have shown that over expressing SOX2 in ESCC cells promote proliferation and *in vivo* tumor growth [12,13]. However, the role of SOX2 in ESCC metastasis has not been completely elucidated. This study was conducted to explore the function and role of SOX2 in EMT, a crucial step in cancer metastasis. We found that SOX2 over expressing Eca-109 cells promoted the processes of migration/invasion and EMT. Moreover, our results showed that SOX2 over expression in Eca-109 cells was correlated with elevated expression of Slug and STAT3/HIF-1α signaling controlled the expression of Slug and thereby affected the process of metastasis, at least in part.

The process of EMT was first recognized as a feature of embryonic development, and it has been found to play a crucial role in cancer progression. As a result, the cells lose polarity and thereby they are rendered with motility to facilitate invasion and metastasis of the tumor. The epithelial trait of cell to cell adhesion is replaced with invasive mobility, a characteristic of mesenchymal cells [39]. The association of EMT with SOX2 was first reported by Lin *et al.* [40], who showed that silencing of SOX2 in colorectal cancer cells induced the mesenchymal-to-epithelial transition (MET), a process reciprocal to EMT. Subsequent *in vitro* studies with breast, prostate and laryngeal cancer cells also showed that SOX2 over expression promoted EMT [41,42]. Consistent with these studies, our results demonstrating the morphological and molecular changes characteristic of EMT have suggested that SOX2 over expression in ESCC cells promoted EMT.

Slug is a transcription factor which is activated during EMT process and its elevated expression is directly associated with the advancement of tumor grade, metastasis and poor prognosis in a variety of human malignancies [43,44]. In this study, we also found that SOX2 over expression was correlated with elevated expression of Slug in Eca-109 cells. In addition, in SOX2-over expressing cells, the knockdown of Slug led to a significant suppression of cell invasion and EMT, indicating the role of Slug in promoting SOX2-induced metastasis, at least in part. Our findings are in line with previous studies, which reported that SOX2 mediated up-regulation of Slug by binding to Slug promoter in hepatocelllular carcinoma and pancreatic cancer cells [30,45]. In addition, we evidenced that inhibition of STAT3/HIF-1α signaling with siRNA significantly suppressed the expression of Slug, suggesting the implication of STAT3/HIF-1α signaling in the SOX2-mediated modulation of Slug expression in Eca-109 cells.

Recently, STAT3/HIF-1α has been shown to play a key role in EMT process [46,47]. In addition, SOX2 has been reported to modulate the signaling upstream of STAT3 pathway [48,49]. In complete agreement, our results suggest that SOX2 modulated the processes of cell invasion and EMT, at least in part via the activation of STAT3/HIF-1α signaling. Whereas our study showed that in ESCC, SOX2 exerted a regulatory action upstream of STAT3/HIF-1α, another report indicated the action of SOX2 downstream of HIF-1α in cancer cells other than ESCC [50]. Similarly, STAT3 has been reported to be activated upstream of SOX2 in breast and lung cancer stem cells [51].

## 4. Experimental Section

### 4.1. Cell Culture

Eca109 cell line was obtained from the Cell Bank of the Chinese Academy of Sciences (Shanghai, China). The cells were cultured in Roswell Park Memorial Institute (RPMI)-1640 or Dulbecco’s modified eagle media (Wisent Inc., Quebec, QC, Canada) supplemented with 10% fetal bovine serum (Wisent Inc.) and 1% penicillin-streptomycin (Invitrogen of Thermo Fisher Scientific Inc., Waltham, MA, USA) in a humidified 5% CO_2_ atmosphere at 37 °C.

### 4.2. Generation of Plasmid Constructs and Establishment of SOX2-Ovexpressing Cell Line

The SOX2-coding DNA sequences were obtained by RT-PCR and cloned into pCMV vector (Beyotime Institute of Biotechnology, Shanghai, China) as previously described [23]. The resulting plasmid was named pCMV-SOX2. Eca109 cells were transfected with pCMV-SOX2 or pCMV vector as control. Two days after transfection, G418 was added to the cells for selection of the clones stably transfected with Eca109/pCMV-SOX2 or Eca109/pCMV. The resulting cells stably expressing SOX2 or control vectors were selected as Eca109/pCMV-SOX2 and Eca109pCMV, respectively.

### 4.3. Quantitative RT-PCR (qRT-PCR)

Total RNA was extract from the cells using RNA simple Total RNA Kit (TIANGEN Co., Beijing, China) and 3 µg was converted into cDNA using the High Capacity cDNA Archive Kit (Applied Biosystems, Foster City, CA, USA). The primers were synthesized based on the published sequence [19,20]. For each PCR reaction, a master mix including SYBR GREEN mastermix (Solarbio Co., Beijing, China), forward primer, reverse primer, and 10 ng template cDNA was prepared. The PCR conditions were 5 min at 95 °C followed by 40 cycles of 95 °C for 30 s, 60 °C for 30 s, and 72 °C for 30 s. Data were analyzed using the comparative Δ*C*_t_ method (ABPrism software, Applied Biosystems, Foster City, CA, USA) using GAPDH as an internal normalization control.

### 4.4. Western Blotting

The cell lysates (30–50 μg) were mixed with 6× sample buffer, boiled for 5 min, electrophoresed in 10% sodium dodecyl sulfate polyacrylamide gel and then transferred to PVDF membranes. The membrane was then blocked in PBS containing 5% bovine serum albumin (BSA) for 1 h at room temperature. The blocked membranes were incubated overnight at 4 °C with specific primary antibodies in Tris-buffered saline. After washing, the membranes were incubated with HRP-conjugated secondary antibodies (Beyotime Institute of Biotechnology, Shanghai, China). ECL detection reagent (7Sea Biotech., Shanghai, China) was used according to manufacturer’s instructions and the protein signals were detected.

### 4.5. Transwell Migration Assay

Transwell chambers (polycarbonate filters of 8-m porosity, Corning, NY, USA) were used in this test. The bottom chamber was filled with culture medium containing 20% FBS. Cells were suspended at a density of 5 × 10^4^ cells/mL in serum-free medium and 1 × 10^4^ cells were plated in the upper chamber. The cells were removed from the upper chamber by a cotton swab following 24-h incubation. Cells penetrated through and attached to the bottom of the filter were fixed with 4% formaldehyde solution for 20 min then stained with 0.1% crystal violet for 5 min. The cells were observed and imaged under a 20× objective of a phase-contrast microscope (Motic China Group Co., Xiamen, China). The number of cells attached to the bottom of filters was counted under a 200× magnification. An average of cells counted in five fields was calculated and each result represents an average of three individually conducted experiments.

### 4.6. Invasion Assay

Plates with 24-well Transwells coated with Matrigel (8-μm pore size; BD Biosciences, San Jose, CA, USA) were used for the cell invasion assays [24]. Equal numbers (1 × 10^5^) of untransfected cells and cells stably transfected with pCMV-N-His-SOX2 or pCMV-N-His were plated in separate wells. Cells were starved overnight in serum-free medium, trypsinized and washed three times in DMEM containing 1% FBS. A total of 1 × 10^5^ cells were then resuspended in 500 μL DMEM containing 1% FBS and added to the upper chamber, while MEM with 10% FBS was added to the lower chamber as chemoattractant. For controls, the medium containing 1% FBS was added to the lower chamber. After 24 h of incubation, the Matrigel and the cells remaining in the upper chamber were removed by cotton swabs. The cells on the lower surface of the membrane were fixed in formaldehyde and stained with hematoxylin staining solution. The cells were photographed and counted in at least five randomly chosen microscopic fields (200× magnification).

### 4.7. Knockdown of STAT3, HIF-1α and Slug

Sense and antisense oligonucleotides targeting STAT3 were synthesized according to the sequences published previously [25]. For silencing, the HIF-1α-targeted siRNA was obtained from Origene (Rockville, MD, USA.). Human Slug siRNA was purchased from Invitrogen (Carlsbad, CA, USA). Nonspecific siRNA, which has no target in the human transcriptome, was used as a negative control. One day before transfection, the cells were plated in a 96-well culture plate at a density of 5 × 10^3^ cells/well and allowed to reach 30% confluence by incubating for 24 h. The transfection mixture containing siRNA and Lipofectamine 2000 (Invitrogen, Carlsbad, CA, USA) was kept for 20 min at room temperature, and then it was added to the cells. The cells were then incubated for 6 h at 37 °C in a humidified atmosphere containing 5% CO_2_. Subsequently, the cells were washed with PBS and maintained in the culture medium for 48 h before measuring the expression of the transcription factors by Western blotting.

### 4.8. Statistical Analysis

The data are expressed as Mean ± SD of three separate experiments, each performed in quadruplicate unless otherwise stated. The Student’s *t* test was used to evaluate if the difference between two groups was significant and One Way ANOVA with *post hoc* test was used for comparison between three or more groups. *p* < 0.05 was considered as statistically significant.

## 5. Conclusions

In summary, we have demonstrated that an over expression of SOX2 led to ESCC metastasis characterized by migration, invasion and EMT. These processes involved SOX2-induced up-regulation of Slug expression via the activation of STAT3/HIF-1α signaling. These findings emphasize the usefulness of SOX2 as the potential therapeutic target for the treatment of LSCC. However, further studies are needed to completely understand the oncogenic role of SOX2 in ESCC.

## Supplementary Materials

Supplementary materials can be found at http://www.mdpi.com/1422-0067/16/09/21643/s1.
