# Supplementary material for: SOX2 Promotes the Epithelial to Mesenchymal Transition of Esophageal Squamous Cells by Modulating Slug Expression through the Activation of STAT3/HIF-α Signaling
Source: Int J Mol Sci. 2015 Sep 8;16(9):21643–57. doi: 10.3390/ijms160921643 (PMC4613272; doi:10.3390/ijms160921643)
Supplement: Supplementary file 1 [file ijms-16-21643-s001.pdf]

Supplementary Information

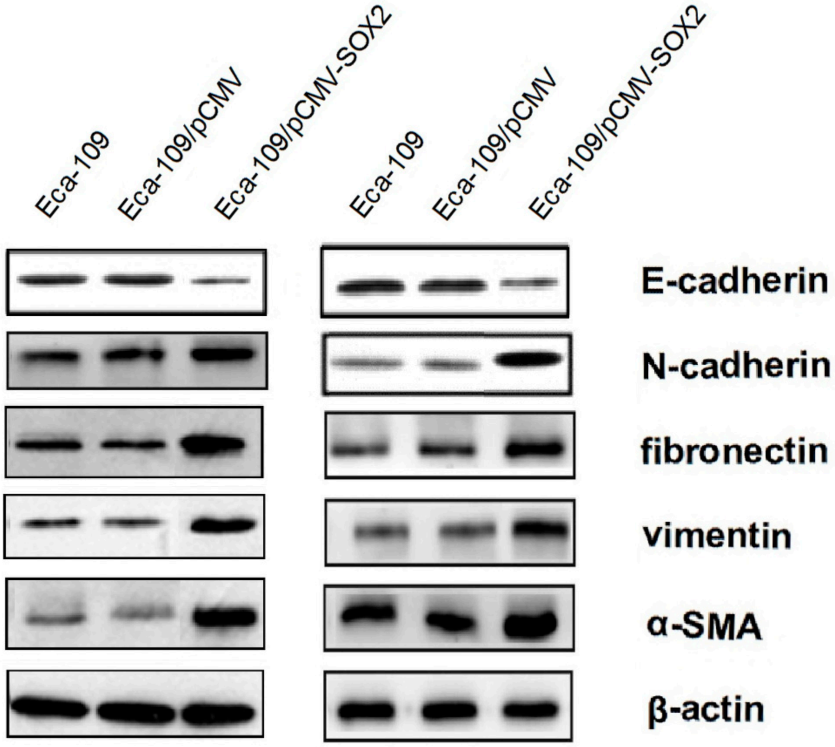

Figure S1. SOX2 over expression promotes the EMT process. The other two Western blot experiments showing the effect of SOX2 on the expressions of epithelial and mesenchymal cells markers (E-cadherin, N-cadherin, vimentin, fibronectin and α-SMA). Supplementary to Figure 2B.

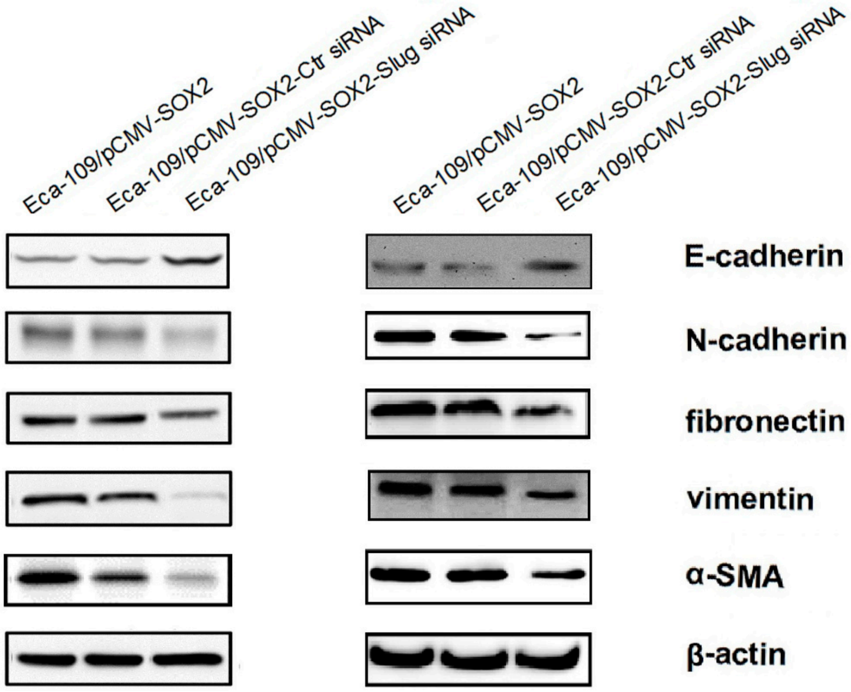

Figure S2. The other two Western blot experiments showing the effect of Slug knockdown on the expression of epithelial and mesenchymal markerssquamous cells markers in SOX2 overexpressing cells. Supplementary to Figure 3C.
